# Florid Bilateral Renal Parenchymal Malakoplakia With Abundant Michaelis-Gutmann Bodies in an Elderly Woman

**DOI:** 10.1016/j.xkme.2026.101370

**Published:** 2026-04-21

**Authors:** Ya-Li Ren, De-Jun Qiu, Xin-Lun Li, Su-Xia Wang

**Affiliations:** 1 Laboratory of Electron Microscopy, Ultrapathologic Center, Peking University First Hospital, Beijing, China; 2 Department of Nephrology, Air Force Medical Center, PLA, Beijing, China

**Keywords:** Electron microscopy, *Enterococcus faecalis*, malakoplakia, Michaelis–Gutmann body, renal parenchyma

## Abstract

Malakoplakia is a rare chronic granulomatous disorder predominantly occurring in immunocompromised patients, with renal parenchymal involvement being exceptionally rare, likely because of dysfunctional macrophage clearance of bacteria‌. Here, we report a case of a 69-year-old woman with elevated creatinine levels and bilateral kidney lesions, but no identifiable immunosuppressive factors. *Enterococcus faecalis* was isolated from urine analysis. Histopathological examination showed nearly complete replacement of renal parenchyma by 3 distinct morphological zones: a granulomatous area rich in CD68^+^ macrophages and giant cells with eosinophilic granular cytoplasm, an inflammatory zone dominated by mixed inflammatory cells, and a collagen-dominant fibrotic zone. Numerous Michaelis–Gutmann (MG) bodies with significant size variation (maximum dimension 187 μm × 96 μm) were observed. Electron microscopy showed abnormally enlarged lysosomes containing fibrillary components, as well as crystalloid or targetoid bodies, representing different developmental stages of MG bodies. After treatment with levofloxacin and prednisone, the patient’s creatinine levels improved, and renal cortical thickness normalized. This case not only demonstrates the histological features of malakoplakia at different stages but also shows the ultrastructural progression of MG bodies, suggesting that malakoplakia should be considered in the differential diagnosis of elderly patients with unexplained kidney function decline.

Malakoplakia is a rare chronic granulomatous disorder characterized by defective macrophage bactericidal activity, most commonly against *Escherichia coli*.^1^^,^^2^ The pathognomonic Michaelis–Gutmann (MG) bodies result from accumulated bacterial debris because of impaired lysosomal digestion. Although malakoplakia has a predilection for the urinary tract, it predominantly affects the bladder and is comparatively scarce in the kidney. The diagnosis of renal parenchymal malakoplakia (RPM) hinges solely on the pathologist’s assessment, as reaching a clinical or radiological conclusion is nearly unfeasible. Diagnostic challenges arise from the condition’s rarity, potential mimicry of renal cell carcinoma, and occasional absence of definitive MG bodies under light microscopy.^3^^,^^4^ This report details an elderly woman patient with advanced-stage RPM, whose distinctive clinicopathological and ultrastructural features provide novel insights into this enigmatic disease.

## Case Report

### Clinical history

A 69-year-old woman with a 20-year history of hypertension was hospitalized for elevated creatinine levels over 1 month. She had no history of autoimmune diseases and had never used immunosuppressive drugs.

One month prior, during evaluation for cough, expectoration, and fever (39 °C), she had elevated white blood cell levels (13.14 × 10^9^/L; normal range 3.5 × 10^9^/L to 9.5 × 10^9^/L; other normal values see Table 1), neutrophil levels (11.50 × 10^9^/L), C-reactive protein levels (199.08 mg/L), blood urea nitrogen levels (20.3mmol/L), serum creatinine (sCr) levels (270.74 μmol/L, 3.06 mg/dL), and procalcitonin levels (19.75 ng/mL) with low albumin levels (27.5g/L). Urine cultures grew *Enterococcus faecalis* twice at a 1-month interval. Antibiotic treatment resolved the fever. Baseline sCr levels could not be retrieved because the patient had no prior renal function test records before the current illness.

**Table 1:** Laboratory Parameters of the Patient at Different Clinical Stages.

Laboratory Parameters	Reference Range	One Month Before Biopsy	Prebiopsy	Two Months After Biopsy
White blood cells (10^9^/L)	3.5∼9.5	13.14	4.20	8.67
Neutrophils (10^9^/L)	1.8∼6.3	11.5	1.55	4.73
C-reactive protein (mg/L)	≤5.0	199.08	1.05	<0.50
Erythrocyte sedimentation rate (mm/L)	≤20.0	Not done	69	Not done
Procalcitonin (ng/ml)	≤0.046	19.75	0.13	0.07
Albumin (g/L)	40.0∼55.0	27.5	37.23	39.15
Uric acid (μmol/L)	143∼339	485	624	326
Serum creatinine (μmol/L)	41.0∼81.0	270.74	215.5	165.2
Blood urea nitrogen (mmol/L)	3.1∼8.8	20.3	12.2	12.7
eGFR (mL/min/1.73 m^2^)	>70	14.9	20.82	28.61
IgG (g/L)	8.7∼17.0	Not done	20.5	13.3
Urine creatine (mmol/L)	2.47∼19.20	1.86	7.09	Not done
Urine microalbumin (mg/L)	≤15.000	132.39	118.60	Not done
Urine transferrin (mg/L)	≤2.000	7.9	4.63	Not done
Urine α1-microglobulin (mg/L)	≤12.000	126.96	74.23	Not done
Urine IgG (mg/L)	≤17.500	33.87	24.90	Not done

Prebiopsy tests showed the following: procalcitonin level, 0.13 ng/mL; erythrocyte sedimentation rate, 69 mm/h; blood urea nitrogen level, 12.2 mmol/L; sCr level, 215.5 μmol/L; estimated glomerular filtration rate (eGFR) 20.82 mL/min/1.73 m^2^; uric acid level, 624 μmol/L; IgG level, 20.5 g/L. Routine blood tests, C-reactive protein levels, and electrolyte levels were within normal limits. Urinalysis revealed the following results: specific gravity, 1.008; pH, 5.0; trace protein; 1 white blood cell/high-powered field; <1 red blood cell/high-powered field; urine creatinine level, 7.09 mmol/L; microalbumin/creatinine ratio, 144.88 μg/mg. In addition, elevated microalbumin, transferrin, α1-microglobulin, and IgG levels were noted. Blood/urine cultures were negative.

Ultrasonography showed thickened bilateral renal cortices (2.2 cm thick) with indistinct corticomedullary junctions, multiple hypoechoic areas, and no pelvicalyceal/ureteral dilation along with a smooth bladder wall. Abdominal computed tomography showed bilateral multiple cysts (largest 17 mm) and thickened perirenal septa. A kidney biopsy was performed to investigate kidney failure.

### Kidney biopsy

#### Light microscopy

**Figure 1:**
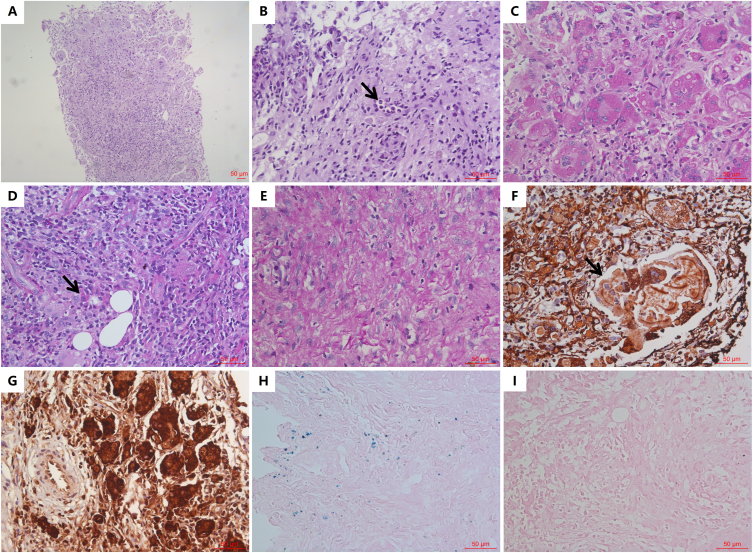
Histopathologic findings. (A) Biopsy shows granulomatous inflammation with large macrophages, multinucleated giant cells, plasma cells, and lymphocytes (H&E, ×100). (B) Neutrophils forming a microabscess (H&E, ×400). (C-E) Periodic acid-Schiff staining shows three lesion zones (×400): phagocytic area with macrophages/giant cells (eosinophilic granular cytoplasm (C); reactive area with mixed inflammatory cells and occasional granular cell (D); collogen-rich fibrotic area with minimal inflammation (E). (F) Silver staining shows intracellular/extracellular round/oval argyrophobic MG bodies with the largest measuring up to 187μm × 96μm (×400). (G) CD68-positive cells (×400). (H) Prussian blue staining with scattered punctate positivity (×400). (I) Negative von Kossa staining (×400). Abbreviations: H&E, hematoxylin and eosin.

Kidney biopsy showed near-complete parenchymal replacement by granulomatous inflammation and fibrosis, extending into perirenal adipose tissue. Only one markedly contracted glomerulus and scattered tubules remained identifiable. Characteristic MG bodies were detected both intracellularly and extracellularly, and their spatial distribution was closely correlated with the 3 distinct histopathological zones of the lesion (ie, phagocytic, reactive, and fibrotic regions; Fig 1).

#### Electron microscopy

**Figure 2:**
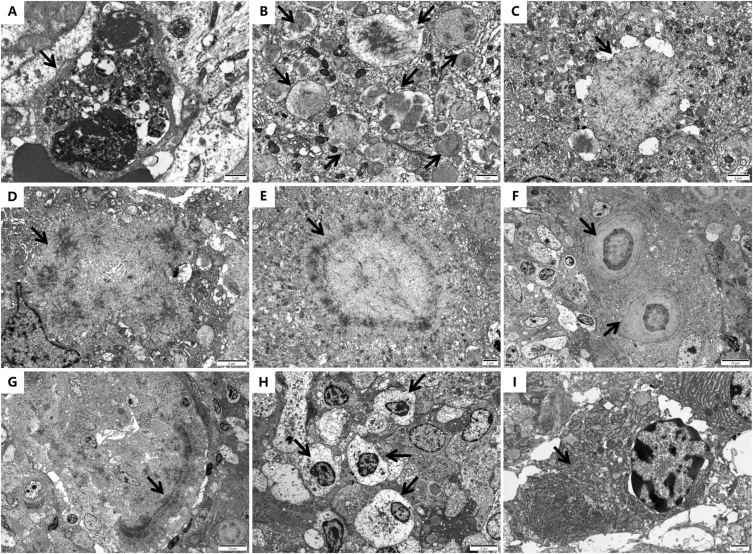
Ultrastructural features. (A) Debris-containing macrophagosome (×12,000). (B) Membrane-bound metamorphic lysosomes with granular/filamentous contents (×12,000). (C-G) MG body maturation sequence: (C) Early filamentous spicules (×6,000); (D) Peripheral ring-forming spicules (×8,000); (E) Near-complete crystalline ring (×4,000); (F) Mature targetoid forms (×1,500); (G) Composite body with discontinuous crystal arcs (×1,500). (H) Degenerating lymphocytes (×3,000). (I) Plasma cell showing endoplasmic reticulum dilation (×10,000).

Electron microscopy showed phagocytes containing numerous macrophagosomes with cellular debris, double-membrane-bound metamorphic lysosomes, and MG bodies (Fig 2). Generally exceeding adjacent mitochondria in size, the lysosomes showed marked variation in dimensions and electron density while predominantly retaining intact membranes containing granular/filamentous material. MG bodies appeared as nonmembrane-bound structures displaying developmental progression from early filamentous aggregates to mature forms. Characteristic targetoid configurations exhibited radial crystal layers with alternating electron density surrounding a central lucent core, whereas variant forms showed peripheral mineralization with central debris accumulation. Lymphocytes predominantly exhibited degenerative changes, including cytoplasmic clearing, organelle depletion, and nuclear hyperchromasia, with occasional apoptotic forms. Plasma cells displayed prominent endoplasmic reticulum dilation. Comprehensive examination showed no bacterial or fungal elements.

### Diagnosis

A diagnosis of renal parenchymal malakoplakia was made.

### Follow-up

The patient was prescribed a 1-month course of oral levofloxacin (0.25 g every day) combined with prednisone (25 mg every day), followed by a systematic tapering of prednisone (2.5 mg reduction every 2 weeks until complete withdrawal). ‌Follow-up imaging showed renal cortical thinning (reduced from 2.2 cm to 1.0 cm) on ultrasonography, whereas concurrent magnetic resonance imaging demonstrated corresponding cyst reduction (Fig S1). At the 2-month mark, renal function tests showed an eGFR of 28.61 mL/min/1.73 m^2^ and sCr level of 165.20 μmol/L (1.87mg/dL), with values remaining stable at 3.5 months (eGFR 28.0 mL/min/1.73 m^2^, sCr level 164.58 μmol/L or 1.86 mg/dL), indicating disease stabilization despite persistent stage 4 chronic kidney disease.

## Discussion

Globally documented malakoplakia cases remained rare, with merely 500 reports by 2020, among which renal parenchymal involvement accounted for no more than 1 out of 5.^5^ This enigmatic condition, although rare, consistently induces acute kidney injury through solitary or multifocal lesions that sometimes simulate malignancies, resulting in nephrectomy decisions driven by diagnostic uncertainty or irreversible renal damage.^6^ The historically dismal prognosis characterized by elevated mortality and compromised renal recovery, particularly in bilateral presentations, underscores the imperative for early histopathological confirmation and precision antibiotic regimens.^7^

A PubMed search (1970-March 2025) identified 138 English-language RPM cases: 20 in grafts and 118 in native kidneys. Grafts were far more susceptible than native kidneys. Among 109 native cases, 106 were analyzed (3 excluded because of extrarenal lesions).^8^, ^9^, ^10^ The native RPM cohort exhibited a wide age range, from a 4-week neonate to an 87-year-old patient, with a mean age of 47.2 years.^11^^,^^12^ The demographic distribution was paradoxical: although the disease was not uncommon in the first decade of life, incidence dropped sharply in the second decade before progressively increasing, peaking in the sixth and seventh decades (Fig. S2). Even immunocompetent elderly patients (as in our case) have higher RPM predisposition, mainly because of age-related phagocytic decline. Reduced lysosomal activity and impaired intracellular killing of pathogens lead to incomplete clearance of invading microorganisms and subsequent accumulation of undigested bacterial debris, ultimately promoting the formation of MG bodies, the pathological hallmark of malakoplakia. A striking 3:1 female predominance emerged alongside frequent bilateral (46.9%) and multifocal (63.6%) involvement, predominantly affecting immunocompromised hosts with diabetes, autoimmune disease, or immunosuppressants; however, some cases, including ours, manifested without classical risk factors.^1^^,^^13^, ^14^, ^15^, ^16^, ^17^

Microbiological profiling showed Gram-negative bacilli, particularly *Escherichia coli*, in 70%-75% cases, with sporadic isolations of *Klebsiella* and *Pseudomonas aeruginosa*.^2^^,^^4^^,^^18^ Our case uniquely identified *Enterococcus faecalis*, previously documented solely in 2 hepatic–renal coinfections.^19^ Bacterial visualization using electron microscopy appears stage dependent, potentially explaining negative results in antibiotic-pretreated specimens like ours.^5^

Pathologically, this case demonstrated 3 exceptional features: exceptionally abundant MG bodies implying profound lysosomal dysfunction; a giant MG body exceeding all published dimensions; and the novel triphasic histology concurrently exhibiting granulomatous, reactive, and fibrotic stages.^20^ These findings collectively represent the morphological evolution of malakoplakia. The absence of MG bodies in fibrotic foci creates diagnostic challenges, and this underscores the critical need for electron microscopy in elderly patients with unexplained renal deterioration.

The ultrastructural pathology exhibited 2 distinctive features. First, macrophages contained abundant abnormal lysosomes characterized by (1) unprecedented numerical predominance over classic lysosomes; (2) substantial size variation (mitochondrial-to-nuclear dimensions); and (3) contents resembling immature MG body precursors, corresponding to eosinophilic cytoplasmic granules on light microscopy. Three pathogenic mechanisms were hypothesized: (1) initial bacterial overload triggering lysosomal hyperactivation; (2) disease-stage correlation (MG body-rich regions indicating active phase); and (3) pathogen-specific effects, whereas our Gram-positive *Enterococcus faecalis* infection lacked comparable ultrastructural data.^19^ Second, we documented the complete morphological spectrum of MG body development, which has rarely been described previously. Consistent with the staging reported by Jung et al,^5^ observed MG bodies represented phagosomal and postphagosomal phases (explaining undetectable pathogens). The maturation process initiates when phagosomal rupture releases star-patterned mineral aggregates into cytoplasm. Subsequent fusion creates polymorphic immature bodies that evolve into targetoid structures with peripheral crystalline rings. Although typical mature bodies display central crystalline cores with concentric laminations, our case confirmed coreless variants also occur. Terminal-stage bodies exhibit fragmented membranes with central matrix mineralization.

Electron microscopy proves indispensable for malakoplakia diagnosis by detecting the following: (1) light microscope-visible mature MG bodies; (2) immature forms across developmental stages; and (3) minute/unmineralized precursors invisible by conventional microscopy. The observed lysosomal abnormalities in macrophages may serve as early diagnostic clues.

In terms of management, literature reports highlight fluoroquinolones as first-line agents for renal malakoplakia and combined surgical intervention even nephrectomy may be employed for severe or refractory cases when necessary.^21^ When the infection has been controlled, glucocorticoid therapy can inhibit interstitial inflammation and improve the prognosis.^22^ Our patient received oral levofloxacin combined with prednisone, leading to stabilized renal function (improved eGFR, reduced sCr levels) and normalized renal cortical thickness on follow-up.

In conclusion, this florid bilateral renal malakoplakia case in an immunocompetent elderly patient with *Enterococcus faecalis* infection advances understanding of MG body ontogeny. It underscores malakoplakia’s inclusion in differential diagnoses for unexplained multifocal renal lesions (granulomatous/fibrotic) in aged individuals, with electron microscopy being crucial for accurate diagnosis, particularly in antibiotic-pretreated cases.

## Figures and Tables

**Supplementary File (PDF):**
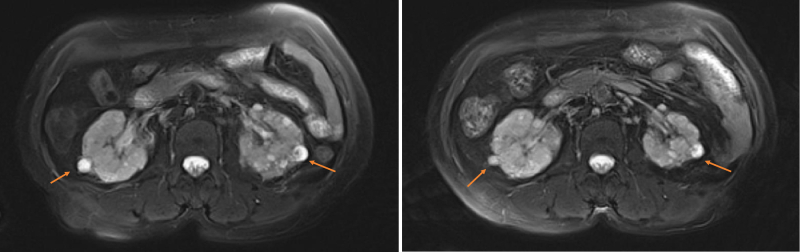
Figures S1.

**Supplementary File (PDF):**
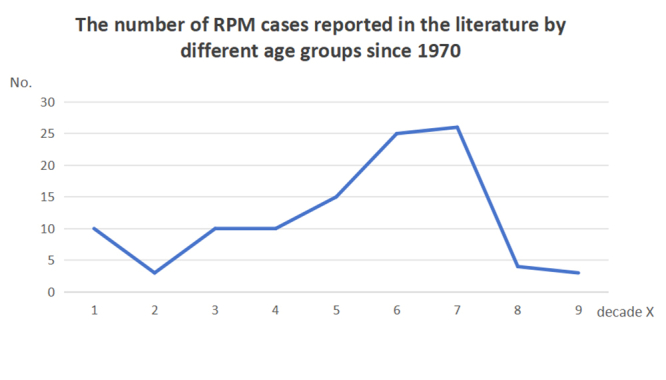
Figures S2.
